# GenAI-Based Digital Twins Aided Data Augmentation Increases Accuracy in Real-Time Cokurtosis-Based Anomaly Detection of Wearable Data

**DOI:** 10.3390/s25175586

**Published:** 2025-09-07

**Authors:** Methun Kamruzzaman, Jorge S. Salinas, Hemanth Kolla, Kenneth L. Sale, Uma Balakrishnan, Kunal Poorey

**Affiliations:** 1 Sandia National Laboratories, Livermore, CA 94550, USA; mkamruz@sandia.gov (M.K.); jsalin@sandia.gov (J.S.S.); hnkolla@sandia.gov (H.K.); klsale@sandia.gov (K.L.S.); 2 Department of Bioengineering, University of Pennsylvania, Philadelphia, PA 19104, USA

**Keywords:** wearables, Generative AI, WGAN, anomaly detection, Data-Driven Digital Twins, verification, validation

## Abstract

Early detection of potential infectious disease outbreaks is crucial for developing effective interventions. In this study, we introduce advanced anomaly detection methods tailored for health datasets collected from wearables, offering insights at both individual and population levels. Leveraging real-world physiological data from wearables, including heart rate and activity, we developed a framework for the early detection of infection in individuals. Despite the availability of data from recent pandemics, substantial gaps remain in data collection, hindering method development. To bridge this gap, we utilized Wasserstein Generative Adversarial Networks (WGANs) to generate realistic synthetic wearable data, augmenting our dataset for training. Subsequently, we use these augmented datasets to implement a cokurtosis-based technique for anomaly detection in multivariate time-series data. Our approach includes a comprehensive assessment of uncertainties in synthetic data compared to the actual data upon which it was modeled, as well as the uncertainty associated with fine-tuning anomaly detection thresholds in physiological measurements. Through our work, we present an enhanced method for early anomaly detection in multivariate datasets, with promising applications in healthcare and beyond. This framework could revolutionize early detection strategies and significantly impact public health response efforts in future pandemics.

## 1. Introduction

Early detection of infectious disease outbreaks is crucial for the containment of their spread. However, current methods for recognizing an outbreak depend on information from healthcare providers such as the number of healthcare visits, hospitalizations, and deaths, which generally lag behind actual accounts of infectious events and their spread [1]. Furthermore, early recognition of potential outbreaks could help in the efficient and targeted implementation of public health interventions such as social distancing guidelines. In recent years, there have been significant advances in numerical and laboratory experiments of virus-laden droplet exhalation dispersal at the individual [2,3] and room level [4], to accurately determine social distancing guidelines. Although these models have advanced our understanding of the dynamics of infectious disease spread, they do not take into account the available information at the population level. Wearable devices, such as smartwatches, offer a promising approach to early outbreak detection by continuously monitoring the health parameters of individuals and populations, making early detection of potential disease outbreaks possible based on the detection of deviations from the baseline physiology when an infection begins. Wearable devices have shown promise in tracking respiratory tract viral infections, including common cold, influenza, and, in recent studies, SARS-CoV-2 [5,6]. In the context of the COVID-19 pandemic, with millions affected globally, wearable devices presented an opportunity for improved infection tracking and early detection at the individual level and adaptive improvement of healthcare and social distancing guidelines at the population level. These devices, already worn by a substantial user base, can measure physiological parameters like heart rate, skin temperature, sleep patterns, and step count. This paper explores both the retrospective and real-time use of wearable device data for early detection of infections in the global pandemic outbreak.

A major task is to identify relevant datasets or instances from raw wearable outputs that are indicative of patient health and whose deviations from normal are indicative of poor health. For example, heart rate is a vital physiological parameter, and abnormal heart rates that span a period can be translated into indicators of various diseases using mathematical models [7]. These abnormal data points, called anomalies, can be measured by existing algorithms in the literature and decision-based systems that can handle the constantly evolving personalized data [8]. However, detection of anomalies in data streams remains a challenge. The first challenge is in evaluating anomalies and distinguishing between true and false positives. Secondly, various statistical and machine-learning-based techniques are employed in anomaly detection based on the field of application, such as bank fraud [9], malware detection, and healthcare. However, the prediction of anomalies in each field is based on trends and signatures which are unique to that field [10]. In the field of healthcare, application of existing anomaly detection methods would require significant re-structuring and unique assumptions [11]. In this work, we used a cokurtosis method-based algorithm for real-time monitoring of the onset of infection using the relationship between heart rate and steps taken daily. The fourth-order standardized moment (cokurtosis) has many merits in univariate [9] and multivariate [12,13] anomaly detection. Using kurtosis as a reliable measure of anomalies, the principal kurtosis vectors (by analogy to principal component analysis vectors) signify the principal directions along which anomalies appear.

The concept of synthetic data generation has gained prominence, aiming to create data that mirrors real-world distributions while mitigating privacy concerns and missing value problems. Various techniques have been employed, including differential privacy [14,15] and machine learning-based approaches [16,17,18,19] that learn from real data and generate synthetic counterparts. One promising approach for generating synthetic data is through Generative Adversarial Networks (GANs), which have been successfully employed in diverse data domains, including images and tabular data [18,20,21]. In this paper, we use a specialized GAN variant tailored for time-series data to synthesize data that fits the distributions of health-related data from wearable devices, including heart rate and step count, with the ultimate goal of predicting the onset of infectious diseases.

The manuscript is organized as follows: in Section 2, we describe the core anomaly detection method, generative AI, and data processing pipelines. In Section 3, we show our experimental results, uncertainty analysis, and one use case of anomaly detection on real data. Finally, we present conclusions in Section 4 with future directions.

## 2. Methodology

The novel contributions of this work focus on an integrated approach to synthetic data generation and a versatile univariate-to-multivariate anomaly detection methodology, which includes

1. The integration of real user wearable data with streaming data, GenAI-WGAN for digital twins and synthetic twins, and fourth-order moment-based anomaly detection offers a new perspective on healthcare data analysis while addressing privacy concerns.
2. Validation of digital twins and synthetic datasets by comparing their statistical signatures with those of real datasets, ensuring the reliability and accuracy of our generated data.
3. Enhancement of real user wearable datasets by augmenting generated digital twins and synthetic datasets, thereby enriching the data available for analysis.
4. Implementing an anomaly detection methodology based on the fourth-order moments of physiological parameters. This approach is beneficial as it can be applied to both univariate and multivariate datasets, accommodating multiple physiological parameters for comprehensive analysis.

**Figure 1:**
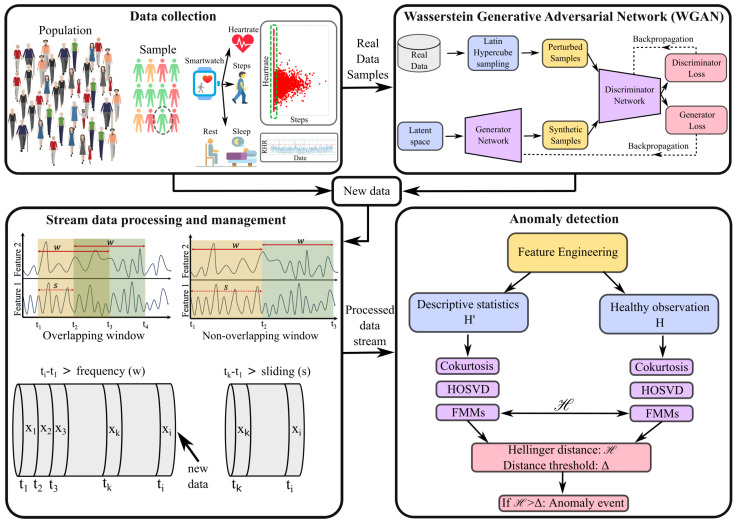
A high-level schematic view of the WGAN and anomaly detection process.

Our anomaly detection process is divided into four main components: (i) data collection, (ii) data generation via Generative Adversarial Networks (GenAI) using WGAN, (iii) stream data processing, and (iv) anomaly detection (see Figure 1). Below are the details of each component.

### 2.1. Data Collection

Health data comprises various measurements of human health vitals, including body temperature, heart rate, blood pressure, blood oxygen level, blood sugar level, blood A1c, blood lipid profile, and more. Modern wearable devices such as Fitbit and Apple Watch continuously monitor some of these vitals, along with tracking activities like steps taken, sleep duration, and more (see top left panel in Figure 1). In this manuscript, we utilized real-world sensor data [22,23] obtained from wearable devices such as Fitbit and Apple Watch. This dataset includes activity data (e.g., steps taken and sleep patterns) and health vital information (e.g., heart rate) for 119 users, collected every 60 s.

### 2.2. Wasserstein Generative Adversarial Networks (GenAI)

Generating synthetic data is challenging due to the lack of information on the true distribution of different features. Generative AI models, particularly Generative Adversarial Networks (GANs), have advanced synthetic data generation using deep neural networks. GANs employ two neural networks that work adversarially. The Generator network creates synthetic data, while the Discriminator network distinguishes between real and synthetic data. Variants of GANs differ in the objectives of the discriminator and generator, as well as in their architectures. Some popular variants include “Vanilla” GAN, WGAN, EB/BEGAN, BiGAN, InfoGAN, ACGAN, VAEGAN, CGAN, and CatGAN [24].

Wasserstein Generative Adversarial Network (WGAN) [25,26] stands out among these variants for its use of the Wasserstein distance, also known as Earth Mover’s distance, measured between two probability distributions. This distance metric helps mitigate common issues in GAN models, such as instability and mode collapse. We would like to point out that we initially attempted to use “Vanilla” GAN and VAE methods for data augmentation. However, we encountered significant challenges: traditional GANs often suffer from instability and mode collapse, while VAEs tend to impose restrictive Gaussian priors on the latent space, limiting their ability to capture the full diversity of the data distribution. Given the limitations we faced with GAN and VAE, we decided to use WGAN. For a visual representation, Figure S1 in the Supplementary Materials depicts the high-level architecture of WGAN, illustrating the functions of the generator and discriminator components within the model. The generator model consists of three layers: (i) input layer, (ii) hidden layer, and (iii) output layer. The number of nodes in the input layer depends on the number of input data features. We used noise data as input, where the user defines the number of features. In our model, we used 100 features. The hidden layer consists of 256 nodes (neurons), with LeakyReLU as the activation function (α=0.2). The number of nodes in the output layer depends on the number of features of the real data. Our dataset consisted of two parts: heart rate and steps. The tanh activation function is used in this layer. The discriminator, like the generator, consists of three layers. The number of neurons in the input layers depends on the number of features in the real data, which is two for our dataset. The hidden layer has the same structure as the generator, and the output layer is a regression layer that consists of one neuron that generates real-valued data. The generator model ($g_{\theta }\left(z\right)$) produces synthetic data from a random sample (latent data), which is drawn from a normal distribution (z∼N(0,1)), and both real and synthetic data have the same number of observations but vary in the number of features. The dimension of synthetic data is the same as the dimension of real data. The discriminator aims to maximize the difference between real and synthetic data, while the generator aims to minimize this difference. The min–max objective between the Generator (*G*) and discriminator (*D*) is

(1) $$\min_{G}\max_{D\in D}\underset{x∼{I\;P}_{r}}{E}\left[D\left(x\right)\right]+\underset{\bar{x}∼{I\;P}_{g}}{E}\left[D\left(\bar{x}\right)\right].$$

Here, ${I\;P}_{r}$ is the real data distribution, ${I\;P}_{g}$ is the model distribution, and D is the set of 1-Lipschitz functions. $\bar{x}=G\left(z\right)$ is the generator output with input z∼P(z) sampled from a noise distribution that follows a Gaussian distribution. A weight clipping mechanism, which limits the discriminator weights to [−0.01, 0.01], is enforced to ensure the 1-Lipschitz function.

### 2.3. Stream Data Processing

Stream data processing of wearable data is a powerful approach that enables real-time monitoring and analysis of health and wellness, facilitates the prediction of adverse changes in health, and ultimately provides information for decision-making in healthcare. Papadimitriou et al. [27] used the streaming of multidimensional time series data to detect patterns without employing any buffer to store data or conducting pair-wise stream data comparisons. Zhou et al. [28] used non-overlapping intervals to compute the similarities between two intervals for anomaly detection. Yufeng et al. [29] studied anomaly detection using non-overlapping windows of time series data by predicting data for each window to detect anomalies that do not fall within the predicted data in a specific confidence interval. Our sliding window-based anomaly detection method is closely aligned with the study of Dasgupta and Forrest [30], and Keogh et al. [31]. Dasgupta and Forrest proposed sliding window-based anomaly detection, using two other time series datasets named detection and reference to detect anomalies within a window. The detector was trained using a reference set to detect anomalies in the input data. In our sliding window-based anomaly detection method, we randomly selected from previously distributed data instead of training on the reference data. Keogh et al. [31] also proposed a sliding window-based anomaly detection method using a reference set where the frequency of occurrence is a key factor in detecting an anomaly. We used a data transformation pipeline to compute the probability distribution of both input and reference data. An anomaly event is detected using a distance-based metric between the probability distributions of the real and reference data. Moreover, our method can store and identify anomalies in real-time by dynamically adjusting the size of the data queue, which is described as follows.

We used a variable-length time-sensitive queue (Q) to capture time series data in real time. This queue stores the data stream for a specific period, called a window (*w*). Based on the length of the time window, (Q) accumulates data and then extracts descriptive statistics from this aggregated data to create latent features. Once the latent features are extracted, the queue shrinks to prepare for the following data timestamp. The shrinking period is called sliding (s) and will not exceed the window length. This means the queue retains the data for a shorter duration after feature extraction, making room for new incoming data. Figure S2 in the Supplementary Materials shows the visual representation of the sliding window. Moreover, Algorithm S1 (in the Supplementary Materials) presents the pseudo-code to process the stream of wearable health data. The extracted latent features represent an aggregate representation of the data stream throughout the time window. Our anomaly detection process analyzes these features to determine if there are any anomalies present in the data stream during this time window. This sliding-window mechanism using (Q) allows for efficient processing of the continuous health data stream, enabling the detection of anomalies and timely responses to potential health issues.

### 2.4. Anomaly Detection

Anomaly detection in healthcare data involves identifying abnormal behavior in one or more physiological events, such as heart rate, blood pressure, and oxygen level. Detecting anomalies in healthcare data is challenging due to variations in individual tolerance levels for physiological events, which can depend on factors like age, gender, comorbidity, etc.

Our anomaly detection technique is based on the principle that higher-order joint moments reliably contain information about outlier samples in multivariate data, successfully demonstrated for scientific simulation data [12,13]. Specifically, factors of cokurtosis, the fourth-order joint cumulant, can be inspected for the presence of outliers. This method has been compared to other existing algorithms in the past, such as the Local Outlier Factor method [12,32], demonstrating excellent performance. It has also been successfully deployed in an exascale computing environment, ensuring that our approach is scalable and capable of handling large volumes of data [33]. Cokurtosis is particularly advantageous for multivariate data, as it captures higher-order dependencies that are often overlooked by traditional methods. In contrast to LSTM, which excels in sequential data but may struggle with noise and dynamic baselines due to its complexity [34], cokurtosis provides a more interpretable framework that is robust to noise. Isolation forest [35], while effective for outlier detection, may not adequately address the temporal dynamics inherent in the wearable sensor data. Cokurtosis provides a more interpretable framework that is robust to noise and suitable for real-time processing, making it a compelling choice for our study.

We require at least two features (variables) for this method. Sometimes, having too many features can be challenging due to various limitations. Some issues could be related to data collection, experimental setup, etc. We compute descriptive statistics (mean, median, minimum, maximum, etc.) to derive more features when we have only one feature. Let $X\in R^{\left(n\;\times \;m\right)}$ be a dataset where *n* is the number of observations and *m* is the number of features. X can be represented as a set of column vectors, $X=\{x_{i}\in R^{\left(n\;\times \;1\right)}\;\forall i\in \{1,\ldots ,m\}\}$. The cokurtosis joint cumulant tensor $T\in R^{\left(n\;\times \;n\;\times \;n\;\times \;n\right)}$ is defined, using index notation, as

(2) $$T_{ikjl}=E\left(x_{i},x_{j},x_{k},x_{l}\right)-E\left(x_{i},x_{j}\right)E\left(x_{k},x_{l}\right)-E\left(x_{i},x_{k}\right)E\left(x_{j},x_{l}\right)-E\left(x_{i},x_{l}\right)E\left(x_{j},x_{k}\right),\;i,j,k,l\in \left\{1,\ldots ,m\right\},$$

where E is the expectation operator. Instead of examining the cokurtosis tensor in its entirety, the statistical information within can be further distilled by factorizing it. Being a symmetric fourth-order tensor (its entries are invariant to permutation of indices), it can be factorized using a variety of tensor decomposition techniques, but following the original work [12], we perform a simple higher-order singular value decomposition (HOSVD) of T and Obtain the singular values and singular vectors. To illustrate the capabilities of the methodology, Figure S4 in the Supplementary Materials shows four synthetic, uncorrelated bivariate Gaussian datasets with zero mean and variances [1.0,0.2], which are then mixed by rotation, along with a few sample outliers in different locations. In each panel, we can see that the principal co-kurtosis vectors align in the direction of the outliers. We can then convert the resulting “principal” kurtosis vectors and values into feature moment metrics (FMMs). If $\left(\lambda_{k},{\hat{v}}_{k}\right)$ are singular value/singular vector pairs resulting from HOSVD of the cokurtosis tensor, the FMMs are defined as

(3) $$F_{i}=\frac{\sum_{k=1}^{n}\lambda_{k}{\left({\hat{e}}_{i}\cdot {\hat{v}}_{k}\right)}^{2}}{\sum_{k=1}^{n}\lambda_{k}},\;\;\;i=\left(1,\cdots ,n\right),$$

where $\left({\hat{e}}_{i}\cdot {\hat{v}}_{k}\right)$ is effectively the *i*-th entry in the *k*-th singular vector, ${\hat{v}}_{k}$.

Intuitively, $F_{i}$ denotes the kurtosis fraction in the data attributable to the *i*-th feature. Accordingly, the FMM is a vector of size *n* and sums to unity, thereby having the flavor of a discrete distribution. Bottom-right panel of Figure 1 depicts the anomaly detection algorithm, while Algorithm S2 (in Supplementary Materials) shows the pseudo-code to detect anomalies on a stream of wearable health data.

The anomaly detection leverages the principle that the FMM vector of a dataset with anomalies will likely differ from that of a normal dataset, quantified by a simple divergence metric called Hellinger distance. Let *x* and *y* be two random variables derived from two discrete probability distributions x∼P(X) and y∼P(Y). The Hellinger distance is a divergence metric (a measure of difference) between P(X) and P(Y) and computed as $H\left(x,y\right)=\frac{1}{\sqrt{2}}\times {\left|\left|\sqrt{x}-\sqrt{y}\right|\right|}_{2}$. We compute the Hellinger distance between the distributions of FMM computed from the streamed data ($d_{1}$ in Algorithm S2 in the Supplementary Materials) and the reference healthy population ($d_{2}$ in Algorithm S2 in the Supplementary Materials). A threshold (Δ) on the Hellinger distance H is used to detect an anomaly event (H>Δ).

#### Anomaly Detection in Health Data

Understanding a person’s health profile during a healthy period is crucial in detecting an anomaly event. For example, knowing the maximum blood pressure, maximum resting heart rate (RHR), and minimum oxygen levels during a healthy period can help identify anomalies. On this matter, the distribution of health profiles during a healthy period is essential but hard to achieve. Hence, we propose an alternative: let α,β be health vitals (heart rate and steps in the present case) with the range of values (min,max) observed among the healthy populations as $\left(\alpha_{l},\alpha_{h}\right)$ and $\left(\beta_{l},\beta_{h}\right)$, respectively. From this range of values, we created a distribution $H=\{\left(\alpha_{i},\beta_{j}\right):\forall \alpha_{l}\leq i\leq \alpha_{h},\forall \beta_{l}\leq j\leq \beta_{h}\}$. The current observation (collected from time-sensitive queue *Q*) of these health vitals $D=(\alpha_{t},\beta_{t})$ is collected and appended with *H* to create a new distribution $H^{\prime }=H\cup D$. We then compute the cokurtosis joint cumulant tensor T for each of these distributions ($H\;\&\;H^{\prime }$), perform HOSVD of cumulant tensors (Equation (2), $T_{1}\;\&\;T_{2}$) and compute the FMMs (Equation (3), $d_{1}\;\&\;d_{2}$). Finally, the Hellinger distance is used to identify the behavioral pattern (normal and anomaly).

### 2.5. Model Evaluation

To evaluate the performance of our anomaly detection technique, we set up a margin ∇ on RHR and another margin Δ on Hellinger distance (H). A patient is considered sick when RHR >∇ and a patient is estimated as sick when H>∇. In population level, we constructed a confusion matrix with the following rules: (i) True Positive (TP): the number of patients with RHR >∇ and H>Δ, (ii) True Negative (TN): the of patients with RHR ≤∇ and H≤Δ, (iii) False Positive (FP): the number of patients with RHR ≤∇ and H>Δ, and (iv) False Negative (FN): the number of patients with RHR >∇ and H≤Δ. We evaluate our model using the “$F_{1}$ score” and False Negative Rate (FNR) as performance metrics. We aim to maximize the “$F_{1}$ score” and minimize the FNR.

## 3. Results and Discussion

In our experiment, we collected time-series real-world wearable sensor data ($D_{r}$) from sources such as [22,23], known as “ground truth”. This data is recorded at regular intervals, providing a comprehensive view of an individual’s health during COVID-19 over three months.

**Figure 2:**
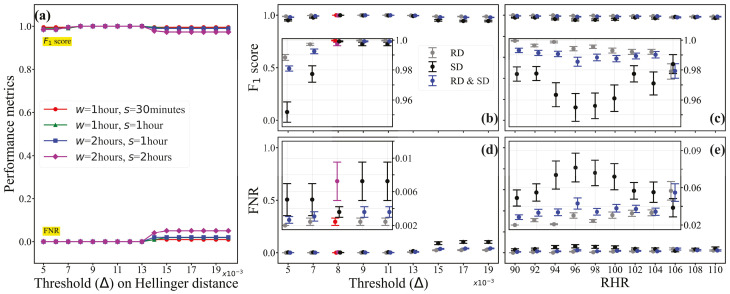
The experimental results of (**a**) the model performance for different window and sliding selections, and (**b**–**e**) the uncertainty of the model’s performance (measured by $F_{1}$ score and FNR (False Negative Rate)) using both real and synthetic twin data. In (**a**), *w* refers to the window length, and *s* refers to the sliding length. In (**b**), RD refers to real data (i.e., $D_{r}$), and SD refers to synthetic data (i.e., $D_{s}$). A different color scheme (red for RD, magenta for SD and black for RD and SD) is used (in (**b**,**d**)) to indicate the threshold where we observed the highest $F_{1}$ score and the lowest FNR.

As described in stream data processing methodology, our analysis involves a sliding-window queue (*Q*) with two key parameters: window length (‘*w*’) and sliding length (‘*s*’). We explore how these parameters affect model performance by considering two types of windows: overlapping (s<w) and non-overlapping (s=w). A schematic representation of these two types is shown in the bottom-left panel of Figure 1. For overlapping windows, we test two configurations: (i) a 1-h window with 30-min sliding and (ii) a 2-h window with 1-h sliding. For non-overlapping windows, we also test two configurations: (i) a 1-h window with 1-h sliding and (ii) a 2-h window with 2-h sliding. In our analysis, we assess the performance of our anomaly detection model across different Hellinger distance thresholds (0.005≤Δ≤0.02) using dataset $D_{r}$ for all four combinations of window and sliding selections. We use a cutoff margin of RHR ∇=100, where a patient is classified as sick when RHR >100 and healthy otherwise. Figure 2a shows the “$F_{1}$ score” and FNR against threshold Δ across different *w* & *s* combinations, which demonstrates the consistent performance of our model across four different window and sliding selections for a real dataset of 111 individuals ($D_{r}$). The $F_{1}$ score remains high, indicating accurate anomaly detection, while the False Negative Rate (FNR) stays low, suggesting few missed anomalies.

We aim to develop a robust anomaly detection method to identify anomalies in individuals with diverse health profiles. RHR varies at the individual level due to factors such as age, gender, and comorbidities. However, it is unclear what range of Hellinger distance should be considered anomalous. To address this, we studied the uncertainty of our model’s performance (with a 90% confidence interval) across a range of RHR values (90≤∇≤110) and Hellinger distance thresholds (0.005≤Δ≤0.02).

Figure 2a demonstrates the consistent performance of our model across four different window and sliding selections for a real dataset of 111 individuals ($D_{r}$). The $F_{1}$ score remains high, indicating accurate anomaly detection, while the False Negative Rate (FNR) stays low, suggesting few missed anomalies. It is worth clarifying that our dataset comprises time series health profiles for 111 users, with data collected every minute over an average period of four months. This results in approximately 172,000 observations per user. Consequently, the combined dataset contains around 19.1 million observations in total.

Using 111 real individual user data, serving as the benchmark ($D_{r}$), Figure 2b ($F_{1}$ score vs Threshold (Δ) in grey color) and Figure 2d (FNR vs Threshold (Δ) in grey color) helped us determine a threshold of 0.008 on the Hellinger distance, beyond which an event is considered an anomaly. To find the corresponding cutoff on RHR, we analyzed Figure 2c ($F_{1}$ score vs. RHR in grey color) and Figure 2e (FNR vs RHR in grey color). In Figure 2c, we examined the $F_{1}$ score’s 90% confidence interval against RHR values for thresholds (0.005≤Δ≤0.008). The analysis showed that the model’s performance fluctuated for RHR values below 100 but stabilized thereafter. This led us to select 100 as the cutoff value for RHR. This aligns with the Centers for Disease Control and Prevention’s (CDC) general RHR range of 60 to 100 beats per minute (bpm) [36]. Similarly, Figure 2e displayed the FNR’s 90% confidence interval against RHR for the same thresholds. It revealed fluctuations in the model’s performance for RHR values below 100, followed by a more consistent performance. This alignment with Figure 2c reinforced our choice of 100 as the cutoff value for RHR, corresponding to a Hellinger distance threshold of 0.008. The zoomed-in subfigure reveals the model’s performance consistency up to a threshold of Δ≥0.008, with a slight improvement in FNR for thresholds Δ<0.008.

Our experimental data consists of daily activity and health vitals for 111 real users, among whom 44 were reported sick with a respiratory disease (COVID-19, influenza, etc.). By setting the resting heart rate (RHR) to 100 and the Hellinger threshold to 0.008, our anomaly detection method correctly raises pre-symptomatic warnings for 41 (93%) sick users. Our method outperforms the prior studies conducted by Mishra et al. [7] (63%) and Alavi et al. [8] (80%).

Due to the variability in patients’ health profiles, we cannot limit our analysis by keeping the resting heart rate (RHR) fixed at 100 to detect health anomalies for every individual; instead, we prefer an auto-adapted personalized upper bound for healthy RHR. To achieve this, we continuously observed the health profile for a certain period (24 to 48 h) and selected the 90th percentile of the RHR, which falls within the RHR band ranging from 90 to 110. The choice of the 90th percentile is empirical and aims to reduce false positive warnings.

**Figure 3:**
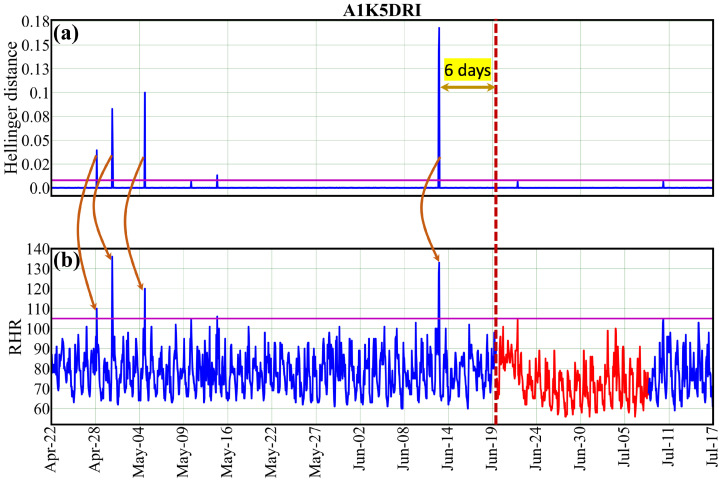
The average resting heart rate (RHR) and Hellinger distance (HD) are plotted over a three-month period for the user “A1K5DRI” (in blue). The period of reported illness due to a respiratory disease is highlighted in red. The purple horizontal line in (**a**) indicates the threshold (Δ=0.008) for the HD, while (**b**) shows the margin for the RHR (∇=105). The brown arrow indicates the HD corresponding to the RHR value.

Next, we intend to evaluate the effectiveness of the threshold values for the Hellinger distance, determined based on $F_{1}$ score and FNR, using real data (ground truth), especially from individuals who are sick (e.g., user ‘A1K5DRI’). Figure 3b illustrates the three-month RHR data, highlighting periods when the user was sick due to a respiratory disease (shown in red). The purple horizontal line indicates the adapted healthy RHR boundary. Figure 3a displays the computed Hellinger distance (HD) using our method. The purple horizontal bar represents the threshold (Δ=0.008) for the Hellinger distance; any spikes in HD that cross this threshold trigger an alert for potential sickness. We have also tested the effectiveness of the threshold values for the Hellinger distance on several additional users, as depicted in the Supplementary Materials. Figure S5 in the Supplementary Materials shows the health profiles for four randomly selected sick users named ‘AOYM4KG’, ‘APGIB2T’, ‘AR4FPCC’, and ‘AQC0L71’. The red vertical dotted line indicates the start of a sick interval. For each user, our anomaly detection method generates a series of alerts before the onset of actual sickness, validating the effectiveness of the chosen threshold value.

Relying solely on a limited amount of real user data is insufficient for developing effective algorithms for anomaly detection at the population level. The scarcity of personalized health data, often constrained by factors such as cost and privacy concerns, poses significant challenges. For machine learning models to make informed decisions, it is essential to have access to a sufficient quantity and quality of data. A larger dataset helps reduce the model’s variance, while high-quality data minimizes bias. However, collecting real health data that satisfies both criteria can be difficult. In this context, synthetic data generation emerges as a viable solution. By creating synthetic data that closely resembles real data (Digital Twin), we can train machine learning models on larger and more diverse datasets, ultimately leading to more robust decision-making processes.

A digital twin (DT), a virtual representation of a real-world object or system, is crucial in healthcare for its ability to replicate a patient’s health status and predict treatment responses. The term DT was formally introduced by Michael Grieves in 2005 within the context of product lifecycle management. By around 2010, NASA and John Vickers further advanced the concept, utilizing it as a virtual model of physical systems [37,38]. Since then, various types of DTs have been proposed in the literature [39,40,41].

In our study, we aim to reproduce or simulate heart rate and steps taken by individuals through a digital representation that incorporates data from real users. This approach aligns with the concept of a functional or mirror twin, which is a specific type of digital twin (DT). According to established definitions, a DT comprises three components: the physical entity, the virtual model, and a real data connection between them. Our work fits within the definition of a functional twin as specified in the referenced literature. Therefore, while we have utilized the term “DT” in our paper, we believe our usage is well-informed by the existing literature and can be understood within that established framework.

We deployed a generative adversarial network, comprising two adversarial networks—named the generator and discriminator—that work together to generate synthetic data resembling real input data. The objective of the generator’s loss function is to estimate the probability distribution of the synthetic data from normally distributed noise (${I\;P}_{G}∼N\left(0,1\right)$). The discriminator’s loss function maximizes the distance between the probability distribution of real data (${I\;P}_{R}$) and generated data (${I\;P}_{G}$). We twisted the objective of the discriminator from maximization to minimization to achieve ${I\;P}_{R}\approx {I\;P}_{G}$, which indicates the closeness of the probability distributions between real and generated data. Additionally, using this approach, we achieve the functional DT of the real data. Using the trained generator model, we created DTs for 111 randomly selected real users.

**Figure 4:**
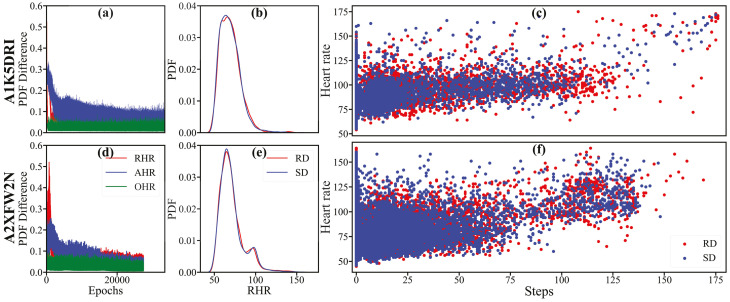
The synthetic data generated using WGAN for two users (‘A1K5DRI’ and ‘A2XFW2N’. (**a**,**d**): The difference in probability density function (PDF) between real data (RD) and synthetic data (SD) for three types of heart rates across each epoch. (**b**,**e**): The PDF of RHR for both RD and SD. (**c**,**f**): The scatter plot of heart rate against step count for both RD and SD. RHR: resting heart rate, AHR: active heart rate, OHR: overall heart rate.

Figure 4a–f illustrate the digital or synthetic twin generated to replicate the profiles of two real users, ‘A1K5DRI’ and ‘A2XFW2N’, using WGAN. Figure 4a,d display the difference (Δ) in the probability density function (PDF) of real user data and their synthetic twin data for resting heart rate (RHR), overall heart rate (OHR), and active heart rate (AHR) at each epoch, representing the Wasserstein distance. The WGAN simulation continued until ΔPDF(RHR) $<7\times 10^{-3}$ or 50,000 epochs, whichever came first. Figure 4b,e present the PDF of real data and their synthetic twin at the final epoch when the WGAN solution converged. Figure 4c,f depict the scatter plot between step counts and heart rates for the respective real and synthetic data of the users. It is evident that the synthetic twin closely mirrors the statistical characteristics of real user data, effectively serving as a “DT”.

We now aim to validate the performance of the digital or synthetic twins ($D_{s}$) generated for the 111 real individual users (black color), and their combination ($D_{r}\cup D_{s}$) (blue color), focusing on $F_{1}$ score and FNR. Figure 2b–e illustrate the uncertainty in $F_{1}$ score and FNR. The black line shows the digital or synthetic twins, and the blue line indicates the combination of real data and their DTs. In Figure 2b, the 90% confidence interval of the $F_{1}$ score against a threshold for RHR values (90≤∇≤110) reveals consistent model performance. To improve visibility, the uncertainty for the first five threshold values is zoomed in and displayed within the figure, showing a slight decline in model performance for thresholds Δ<0.008. In Figure 2d, the 90% confidence interval of the FNR against the threshold shows consistent model performance, with a slight improvement for threshold values Δ<0.008. The findings indicate that our model performs reliably across various thresholds and RHR values, demonstrating its robustness in anomaly detection. All of these validations provide us with the confidence to proceed with generating a small cohort of a synthetic population on a scale comparable to that of a small village.

To augment our dataset to a scale comparable to that of a small village’s population, we introduce random perturbations to real user data (such as heart rate and step count), leveraging this technique in our WGAN for synthetic data generation. This approach enhances our ability to simulate diverse health profiles and improve personalized treatment planning. For this purpose, we employ the Latin Hypercube Sampling (LHS) methodology in conjunction with a random number input [42,43] to introduce statistical noise to the data of real users (see LHS step in top-right panel of Figure 1). Unlike simple random sampling, LHS ensures that random samples are representative of the true variability of the data. It also leads to rapid convergence of estimators for the mean, variance, and population distribution function of the output.

The procedure for generating a synthetic population is as follows: (i) Normalize each feature in the real dataset to the [0,1] range; (ii) Use LHS to draw samples from a normal distribution (z∼N(0,1)), ensuring that the number of samples matches the number of observations in the real data. Generate one sample set per feature (e.g., heart rate, step count); (ii) Pre-multiply the resulting random samples by a perturbation factor ϵ, and add them to the normalized real data of the user. This creates a “perturbed” dataset P=R+ϵL(z∼N(0,1)), where R and L are the real dataset and the random numbers drawn using LHS, respectively; (iv) Scale the perturbed dataset back to the original data range for each feature (e.g., [HRmin,HRmax], [Stepmin,Stepmax]); (v) Ensure that the step count remains above zero in the new perturbed dataset; (vi) Normalize each feature in the perturbed dataset to the [0,1] range; (vii) Provide the perturbed data to the WGAN for synthetic data generation.

We generated over 3000 synthetic datasets (compact group of synthetic population $D_{p}$) using data from only four real users (‘A1ZJ41O’, ‘A0L9BM2’, ‘A45F9E6’, and ‘AXDWDEA’). This represents a collection of more than 30 million synthetic observations. The WGAN was configured to produce a synthetic dataset when the Wasserstein distance between the “perturbed” and “synthetic” datasets for RHR, AHR, and OHR fell below specified thresholds. In our experiments, the WGAN produced a synthetic dataset when ΔPDF(RHR) $<7\times 10^{-3}$, ΔPDF(AHR) $<1\times 10^{-1}$, and ΔPDF(OHR) $<1.5\times 10^{-2}$. We observed that threshold values smaller than specified here did not significantly improve the distribution of sample data.

To ensure the diversity of our synthetic dataset, we implemented the following conditions during the data generation process: (i) We conduct multiple realizations by providing the WGAN with the same real user dataset, but each realization recalculates the LHS perturbations L(z∼N(0,1)) added to the data, which is then multiplied by the perturbation factor ϵ; (ii) Each realization runs for a maximum of 50,000 epochs; (iii) For each realization, we select the synthetic datasets that meet the lower thresholds for ΔPDF(RHR), ΔPDF(AHR), and ΔPDF(OHR), ensuring that a minimum of 1000 epochs separates them.

**Figure 5:**
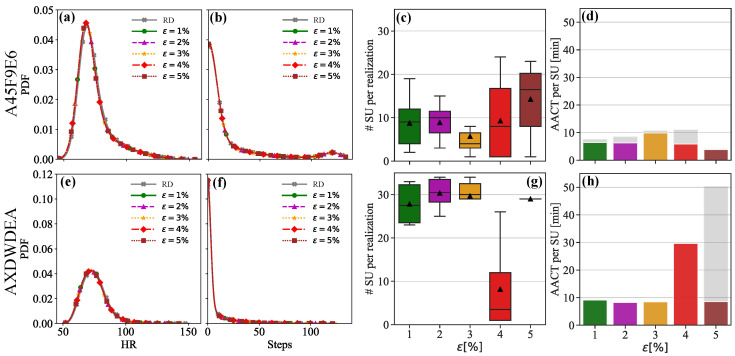
Probability density functions (PDFs) of small cohort of digital twins obtained with different perturbation factors (ϵ) for heart rate (**a**,**e**) and steps (**b**,**f**) for two users (‘A45F9E6’ and ‘AXDWDEA’). (**c**,**g**): Number of synthetic users (SU) generated per realization. (**d**,**h**): Average computing time per synthetic user (AACT).

To assess the performance of the WGAN, we analyze the PDFs for heart rate ((a) & (e)) and steps ((b) & (f)) of two users: ‘A45F9E6’ ((a) & (b)) and ‘AXDWDEA’ ((e) & (f)). The PDFs for real data and synthetic data generated with different ϵ>0% values are compared in Figure 5a,b,e,f. We observe a strong agreement between the PDFs of real and synthetic data, indicating the effectiveness of the WGAN. Figure 5c,g show boxplots of the number of synthetic users (SU) per realization, for different perturbation levels (ϵ). Each boxplot denotes the interquartile range (25th to 75th percentile) with the median (horizontal black line inside box) and mean (black triangle inside box). Our analysis reveals that the WGAN performs efficiently up to a certain level of perturbation ϵ, as illustrated in Figure 5c,d and Figure 5g,h, respectively, for two real user data. For perturbation levels below 3%, our methodology consistently generates a similar number of synthetic datasets regardless of the perturbation factor chosen. However, for perturbation levels above 4%, we observe increased variations in the case of ‘A45F9E6’ and a significant decrease in the number of synthetic datasets produced for ‘AXDWDEA’. This suggests that a perturbation factor less than or equal to 3% is optimal.

Further insights are gained by examining the average computing time per synthetic user (ACT per SU), as shown in panels (d) & (h). This metric is calculated for each ϵ value across all realizations, including those that failed to produce any synthetic data. The gray portion of each bar represents the time wasted by failed realizations. For user ‘A45F9E6’ (top), the ACT per SU ranges between 7.5 and 11 min for ϵ≤4%. However, the contribution to the average computing time from failed realizations is significant for ϵ=4%, indicating inefficiency in data generation. On the other hand, user ‘AXDWDEA’ shows a threefold increase in ACT per SU when increasing ϵ from 3% to 4% (as each user’s physiological signature is unique). This again suggests that a factor ϵ of less than or equal to 3% is optimal for efficient data generation and computational resource utilization.

It is important to note these tests were conducted concurrently, with one ϵ value for one user per computing node, utilizing NVIDIA A100 GPUs. With an average computing time of 500 s per synthetic dataset (for ϵ≤3%), approximately 170 digital twins can be generated in 24 GPU hours. This process can be significantly expedited by running multiple WGANs simultaneously, as computations for each user and ϵ are independent. For example, generating the 3000 digital twins in this study could be achieved by running 18 different combinations of real user datasets and ϵ values on 18 GPU computing nodes for 24 h.

We are interested in validating the synthetic population against the chosen threshold values for the Hellinger distance and RHR, which were determined based on the $F_{1}$ score and FNR, and anomaly detection within small population cohorts. Detecting anomalies in a small cohort of the population is crucial for several reasons: (i) anomalies in a small population can indicate early signs of emerging health issues or outbreaks, enabling timely intervention to prevent the spread of disease or mitigate its impact, (ii) understanding the behavior of anomalies in a small cohort can provide insights into the effectiveness of health interventions or treatments, therein healthcare providers can refine their strategies for better outcomes, and (iii) detecting anomalies in a small population can help in identifying outliers or extreme cases that may require specialized care or attention wherein healthcare providers can intervene promptly and provide targeted care.

**Figure 6:**
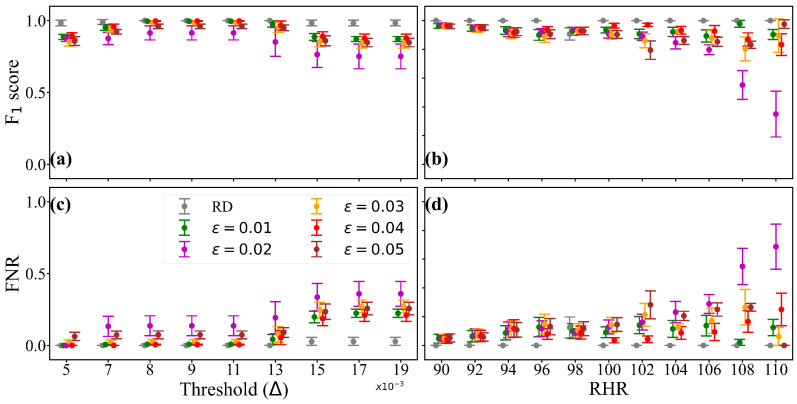
The uncertainty of the model performance for a small cohort of the population created as digital twins. RD: real data. ϵ: % of noise added to RD. FNR: false negative rate. RHR: resting heart rate. (**a**) $F_{1}$ score as a function of threshold Δ. (**b**) $F_{1}$ score as a function of RHR. (**c**) FNR as a function of threshold Δ. (**d**) FNR as a function of RHR.

For our next set of experiments, we randomly selected four real users (‘A1ZJ41O’, ‘A0L9BM2’, ‘A45F9E6’, and ‘AXDWDEA’) from the dataset $D_{r}$, along with their corresponding synthetic datasets from $D_{s}$. We then created dataset $D_{p}$ by introducing systematic noise to the data of these four real users. By aggregating these datasets, we create a digital twin of a small town, representing a heterogeneous synthetic population. Figure 6a–d illustrates the uncertainty analysis of our model’s performance, specifically focusing on the $F_{1}$ score and FNR. The close performance of the perturbed data compared to both the real and synthetic datasets validates the effectiveness of our WGAN in producing digital twins that maintain the statistical characteristics of the real data. Furthermore, our anomaly detection technique, based on selected threshold values for the Hellinger distance and RHR, proved to be effective in this small population cohort.

## 4. Conclusions

Our methodology leverages real datasets, corresponding digital twins, and synthetic datasets to help populations navigate the uncertainty associated with anomaly detection thresholds effectively. From our current work, several key conclusions and observations can be drawn:

The integration of techniques—streaming wearable data, GenAI-WGAN for generating digital twins and synthetic populations, and anomaly detection—offers a novel approach that provides a nuanced and accurate understanding of potential health concerns while significantly reducing the false negative rate (FNR). By augmenting the real dataset with GenAI-based digital twins to enhance population size (using the $D_{s}$ dataset), we observed strong concordance in uncertainty analysis compared to performing the same analysis solely on real data. This consistency persisted when generating synthetic datasets for a small village’s population (using the $D_{p}$ dataset), instilling confidence in our generative algorithm, which we intend to leverage extensively to enhance real-time anomaly detection algorithms for larger populations.

Validation of the digital twins and synthetic population through comparisons of their statistical signatures with those of real datasets demonstrated excellent agreement, reinforcing the effectiveness of our approach. Furthermore, our methodology has the potential to transform healthcare data collection while addressing privacy concerns. We also developed a versatile anomaly detection methodology based on the fourth-order moments of physiological parameters, which can be applied across various datasets, from univariate to multivariate, and is compatible with different healthcare data sources, including wearables.

Through this work, we aim to empower individuals and healthcare systems with a more comprehensive and reliable health assessment tool for the early detection of biothreats or pandemics, such as COVID-19. In future work, we plan to implement a multilayered approach using the developed anomaly detection technique for population-level analysis. This includes implementing a hierarchical approach for detecting anomalies at multiple levels of aggregation (e.g., individual, subpopulation, and population levels) to gain a more comprehensive understanding of health trends and anomalies. Additionally, integrating the anomaly detection technique with epidemiological models could help in understanding the spread of diseases and predicting future outbreaks based on anomalous patterns.

While this study provides valuable insights into the development and validation of a statistically-based anomaly detection framework, we acknowledge that the evaluation of user experience is a crucial aspect in healthcare applications. Unfortunately, the open-source dataset used in this research did not contain user experience data or trust in alerts. Conducting a comprehensive user experience study for the method presented in this paper would require additional resources and infrastructure that are beyond the scope of this proof-of-concept work. We recognize the importance of this evaluation and plan to address user experience in future studies to enhance the practical applicability of our methods.

## Data Availability

The software codes and dataset used in this study are available at https://doi.org/10.11578/dc.20240703.2 (accessed on 7 June 2024). The de-identified data used in this study can be downloaded from the repository (https://storage.googleapis.com/gbsc-gcp-project-ipop_public/COVID-19/COVID-19-Wearables.zip (accessed on 23 May 2023)).

## Supplementary Materials

The following supporting information can be downloaded at: https://www.mdpi.com/article/10.3390/s25175586/s1, Figure S1: The architectural block of WGAN with generator and discrimination models; Figure S2: Sliding window mechanism for processing streaming data; Figure S3: The implementation of the sliding window using a queue Q; Figure S4: Visual explanation of co-kurtosis vectors; Figure S5: Anomaly warnings; Figure S6: Edge computing; Table S1: Comparison between edge and cloud computing.; Algorithm S1: Sliding window mechanism; Algorithm S2: Anomaly detection algorithm.

## Abbreviations

The following abbreviations are used in this manuscript:

WGANs	Wasserstein Generative Adversarial Networks
DT	Digital twins
HOSVD	Higher-order singular value decomposition
FMMs	Feature moment metrics
OHR	Overall heart rate
AHR	Active heart rate
RHR	Resting heart rate
FNR	False negative rate
CDC	Centers for Disease Control and Prevention’s
bpm	Beats per minute
HD	Hellinger distance
LHS	Latin Hypercube Sampling
SU	synthetic users
RD	Real data
SD	Synthetic data
ACT	Average computing time
