# Supplementary material for: GenAI-Based Digital Twins Aided Data Augmentation Increases Accuracy in Real-Time Cokurtosis-Based Anomaly Detection of Wearable Data
Source: Sensors (Basel). 2025 Sep 7;25(17):5586. doi: 10.3390/s25175586 (PMC12431508; doi:10.3390/s25175586)
Supplement: Supplementary file 1 [file sensors-25-05586-s001.zip › sensors-3835987-supplementary.pdf]

Supplementary Materials: GenAI-Based Digital Twins Aided Data Augmentation Increases Accuracy in Real-Time Cokurtosis-Based Anomaly Detection of Wearable Data

Methun Kamruzzaman [1], Jorge S. Salinas [1], Hemanth Kolla [1], Kenneth L. Sale [1], Uma Balakrishnan [1,2,*] and Kunal Poorey [1,*]

S1. Wasserstein Generative Adversarial Networks (GenAI)

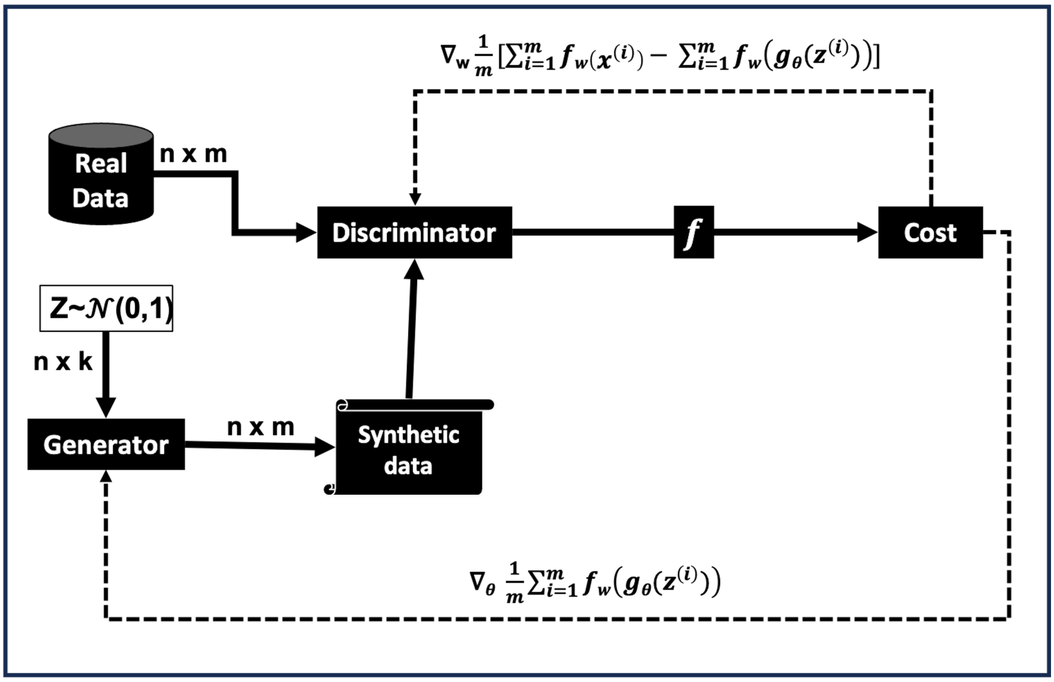

Figure S1. The architectural block of WGAN with generator and discrimination models.

S2. Stream data processing

We collected vital health information from health sensors, which provide data at short intervals. The following sliding-window-based mechanism is established to handle the stream of health data.

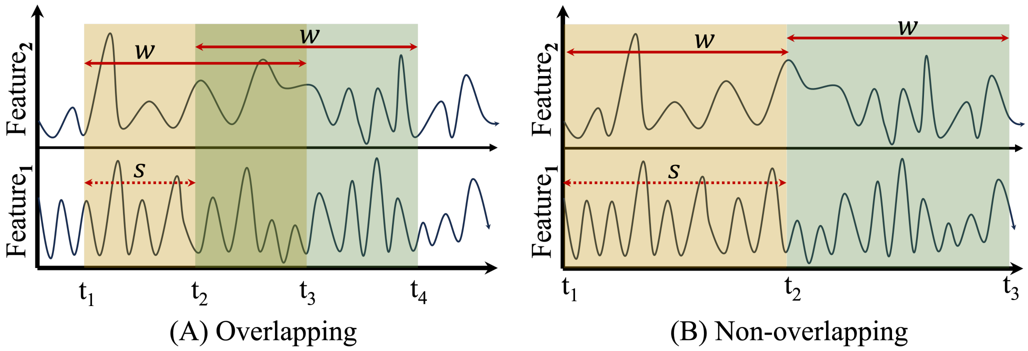

Figure S2. Sliding window mechanism for processing streaming data.

1. We developed a one-hour window to observe health data and extract descriptive statistics (mean, median, etc). To hold this data, we implemented a time-sensitive queue Q that expands up to a window size (Figure S3(A)).
2. When Q size is the same as the window size, we extracted descriptive statistics of all the data within the queue against the start date–time of the window.
3. We remove all the elements from the queue up to the size of the sliding window (Figure S3(A)).

Algorithm S1: Sliding window mechanism.

```
Enqueue(Q, (t, e)): Add new event e to the end of the queue;
Dequeue(Q): Remove the oldest event from the front of the queue;
Head(Q): First time and event in the queue;
Tail(Q): Last time and event in the queue ;
Minute(Δt): Full minutes in time Δt;
NumberOf(e): Number of events;
IsEmpty(Q): Boolean to check if queue is empty;
Mean([e : (t,e) ∈ Q]): Mean of events in queue;
Minimum([e : (t,e) ∈ Q]): Minimum of events in queue;
AddRecord(D, (μ)): Add mean to dataset.
```

```
Input     : time t, event e, window w, sliding s
Output    : Dataset D
Parameter : A queue, Q.
Enqueue(Q, (t, e))
t_start, e ←Head(Q)
t_end, e ←Tail(Q)
D ← ∅
# Queue contains at least w minutes of data
if Minute(t_end − t_start) ≥ w then
    μ ←Mean([e : (t,e) ∈ Q])
    if NumberOf(e)==1 then
        minV ←Minimum([e : (t,e) ∈ Q])
        D ←AddRecord(D, (μ, minV))
    end
    else
        D ←AddRecord(D, (μ))
    end
    # Move the head to s minutes
    t_start, e ←Dequeue(Q)
    t_next, e ←Head(Q)
    while Minute(t_next − t_start) ≤ s do
        Dequeue(Q)
        if IsEmpty(Q)==True then
            break
        end
        t_next, e ←Head(Q)
    end
end
```

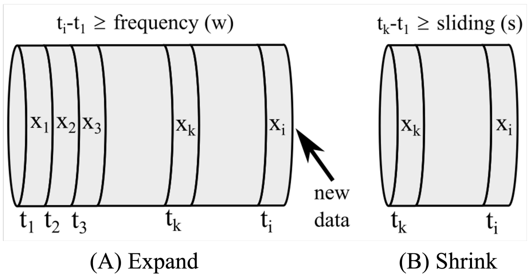

Figure S3. The auto expands and shrinks queue Q is used to implement the sliding window that we showed in Figure S2. The queue expands the data up to the window size and shrinks it to the sliding size.

S3. Anomaly Detection

Figures S4(a)-(d) show synthetic, uncorrelated bi-variate Gaussian datasets with zero mean and variances [1.0, 0.2], which are then mixed by rotation, together with a few sample outliers in different locations. For each panel, the covariance vectors are shown in green (obtained from the SVD of the covariance matrix), while the principal co-kurtosis vectors are shown in blue (obtained from the SVD of the co-kurtosis matrix). First co-variance and co-kurtosis vectors are shown with solid lines, while the second vectors are shown with dashed lines. Focusing on the PCA vectors (in green), we can see that their direction remains nearly unchanged as the location of the anomalous samples is changed. However, the principal co-kurtosis vectors (in blue) align in the direction of the outliers. This behavior illustrates that anomalies result in a change in the distribution of the data, reflecting a change in the orientation of the principal vectors.

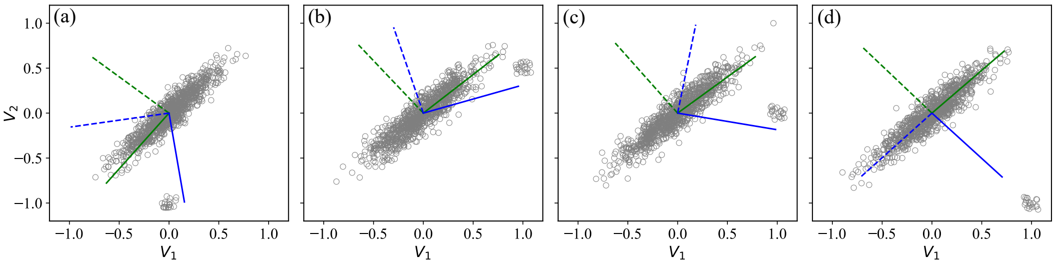

Figure S4. Synthetic, uncorrelated bi-variate Gaussian datasets with zero mean and variance [1.0, 0.25] mixed by rotation, with outliers in different locations. The first (solid line) and second (dashed line) PCA vectors are shown in green, while the corresponding principal co-kurtosis vectors are shown in blue.

Algorithm S2: Anomaly detection algorithm.

```
CoKurtosis(H): Compute cokurtosis of health data H;
SingularVectorDecomposition(T): Perform singular value decomposition of tensor T;
FeatureMomentMetrics(u,s): Compute feature moment metrics using principal-vector
  matrix u and principal-value vector s;
AggregateStreamData(): Add stream data to dataset using Algorithm 1.
-----------------------------------------------------------------------------
Input     : X: The wearable health data
Output    : Anomaly event or not
Parameter : H: The healthy population's health data
Parameter : Δ: Threshold on Hellinger distance.
for (t,e) in X do
    # Using Algorithm 1
    D ← AggregateStreamData(time=t, event=e, window=60, sliding=30)

    if D ≠ ∅ then
        H' ← H ∪ D
        Tensor, T1=CoKurtosis(H')
        u,s,v=SingularVectorDecomposition(T1)
        Density, d1=FeatureMomentMetrics(u,s)

        Tensor, T2=CoKurtosis(H)
        u,s,v=SingularVectorDecomposition(T2)
        Density, d2=FeatureMomentMetrics(u,s)

        Hellinger distance, H=HellingerDistance(d1, d2)
        if H > Δ then
            Anomaly event detected
        end
    end
end
```

S3.1. Anomaly signal detection for sick users

Figure S5 shows the anomaly signals that our system generated for randomly selected four sick users.

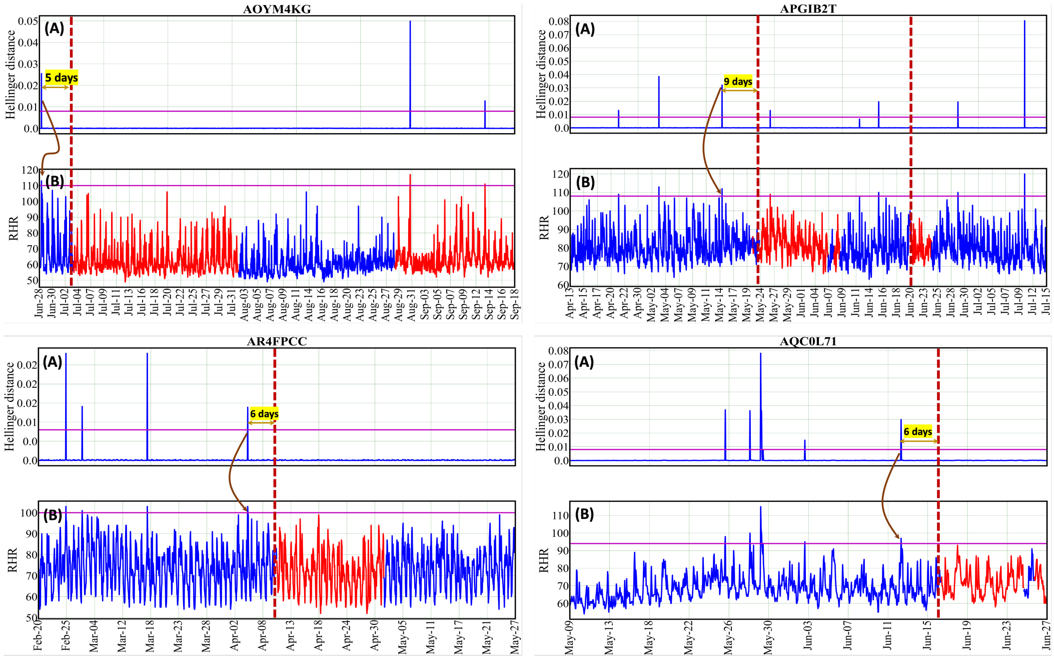

Figure S5. The figure shows the early disease detection capability of our method for randomly selected four sick patients. For each user, (B) shows the RHR for 3 months, where the sick period is marked in red. (A) shows the corresponding Hellinger distance computed using our method.

S4. Edge computing

The edge computing capability of our method is established by developing an iOS app that reads real-time data from an Apple Watch using Bluetooth communication. This app is designed to seamlessly integrate with the Apple Watch, capturing real-time health data such as step count and heart rate. The collected data is then fed directly into our anomaly detection algorithm, which instantly assesses the health status of the user, determining whether they are healthy or experiencing an anomaly. Among all the health vitals received from the Apple Watch, we specifically utilize step count and heart rate for anomaly detection. These metrics are chosen due to their relevance and sensitivity in detecting sudden changes in a person's health status, which could indicate potential health anomalies. The edge computing application offers significant advantages, primarily due to its ability to process data locally on the device. This local processing capability reduces the dependency on a central network, such as a cloud or server, thereby minimizing latency and enhancing the speed of data processing. As a result, the application can perform real-time processing almost instantaneously, which is crucial for AI-driven applications that require immediate responses. By leveraging edge computing, our method ensures faster processing times and reduced run times in the code. This is because the data does not need to be transmitted to a remote server for processing, which can introduce delays. Instead, all computations are performed locally on the iOS device, allowing for rapid analysis and immediate feedback. This capability is critical in scenarios where timely detection of health anomalies can make a significant difference. Figure S6 illustrates the steps involved in the iOS app's process to detect health status. The figure outlines the workflow, starting from the initial data capture from the Apple Watch, through the Bluetooth communication to the iOS device, and finally to the real-time analysis performed by the anomaly detection algorithm. This visual representation helps to clarify the seamless integration and efficient processing enabled by our edge computing approach.

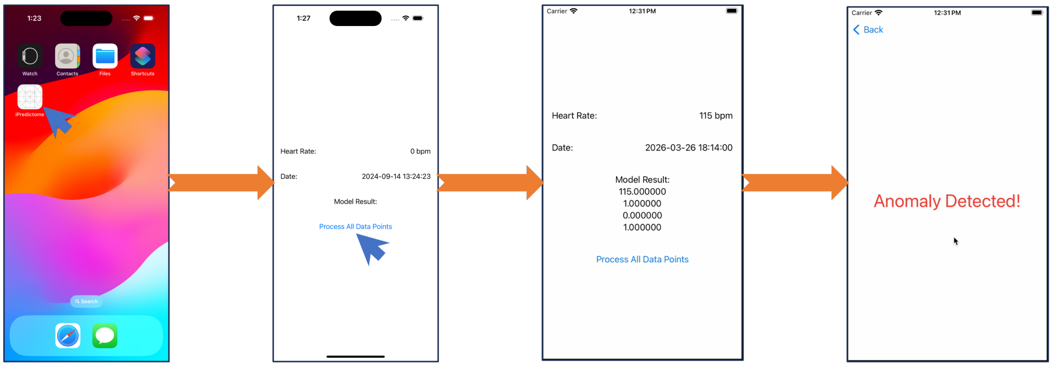

Figure S6. The beta version of the edge computing capability of our anomaly detection method tested on iOS.

Table S1. Comparison between edge and cloud computing.

Component	Edge Computing	Cloud Computing
Synthetic Data Generation	WGAN generate synthetic data locally for immediate use, ensuring low latency	WGAN generates synthetic data in the cloud, allowing for larger datasets and more complex models
Data Streaming	Sliding window queue processes data in real-time on the edge device, minimizing delays	Data is streamed to the cloud for centralized processing and analysis, enabling scalability
Anomaly Detection	Lightweight, higher-order moment-based method detects anomalies on the edge, suitable for limited resources	The same method is applied in the cloud, benefiting from greater computational power for more extensive analysis
Application Development	iPhone app provides real-time anomaly detection for users based on wearable data	Cloud-based analytics support broader insights and data aggregation from multiple users
